# A split-optimization approach for obtaining multiple solutions in single-objective process parameter optimization

**DOI:** 10.1186/s40064-016-3092-6

**Published:** 2016-08-26

**Authors:** Manik Rajora, Pan Zou, Yao Guang Yang, Zhi Wen Fan, Hung Yi Chen, Wen Chieh Wu, Beizhi Li, Steven Y. Liang

**Affiliations:** 1 George W. Woodruff School of Mechanical Engineering, Georgia Institute of Technology, Atlanta, GA 30332 USA; 2 Mechanical Engineering College, Donghua University, Songjiang District, Shanghai, 201620 China; 3 Regional R&D Services Department, Metal Industries Research and Development Center, Taichung, 407 Taiwan, ROC

**Keywords:** Split-optimization, Multiple solutions, Process parameter optimization, Genetic algorithm (GA), Electrochemical micro-machining (EMM)

## Abstract

It can be observed from the experimental data of different processes that different process parameter combinations can lead to the same performance indicators, but during the optimization of process parameters, using current techniques, only one of these combinations can be found when a given objective function is specified. The combination of process parameters obtained after optimization may not always be applicable in actual production or may lead to undesired experimental conditions. In this paper, a split-optimization approach is proposed for obtaining multiple solutions in a single-objective process parameter optimization problem. This is accomplished by splitting the original search space into smaller sub-search spaces and using GA in each sub-search space to optimize the process parameters. Two different methods, i.e., *cluster centers* and *hill and valley splitting strategy,* were used to split the original search space, and their efficiency was measured against a method in which the original search space is split into equal smaller sub-search spaces. The proposed approach was used to obtain multiple optimal process parameter combinations for electrochemical micro-machining. The result obtained from the case study showed that the *cluster centers* and *hill and valley splitting* strategies were more efficient in splitting the original search space than the method in which the original search space is divided into smaller equal sub-search spaces.

## Background

In today’s rapidly changing scenario in the manufacturing industries, optimization of process parameters is essential for a manufacturing unit to respond effectively to the severe competitiveness and increasing demand for quality products in the market (Cook et al. 2000). Previously, to obtain optimal combinations of input process parameters, engineers used a trial-and-error-based approach, which relied on engineers’ experience and intuition. However, the trial-and-error-based approach is expensive and time consuming; thus, it is not suitable for complex manufacturing processes (Chen et al. 2009). Thus, researchers have focused their attention on developing alternate methods to the trial-and-error-based approach that can help engineers obtain the combination of process parameters that will minimize or maximize the desired objective value for a given process. The methods for obtaining these combinations of process parameters can be split into 2 main categories: 1. forward mapping of process inputs to a performance indicator with backwards optimization and 2. reverse mapping between the performance indicators and the process inputs. In forward mappings, first, a model is created between the process inputs and the performance indicators using either physics-based models, regressions models, or intelligent techniques. Once a satisfactory model has been created, it is then utilized to obtain the combination of process parameters that will lead to a desired value of the output using optimization techniques such as the Genetic algorithm (GA), Simulated Annealing (SA), Particle Swarm Optimization (PSO), etc. The desired output can either be to a. minimize a given performance indicator or b. reach a desired level of a performance indicator.

Chen et al. (2009) utilized the back propagation neural network (BPNN) and GA to create a forward prediction model and optimize the process parameters of plastic injection molding. Ylidiz (2013) utilized a hybrid artificial bee colony-based approach for selecting the optimal process parameters for multi-pass turning that would minimize the machining cost. Senthilkumaar et al. (2012) used mathematical models and ANN to map the relationship between the process inputs and performance indicators for finish turning and facing of Inconel 718. GA was then used to find the optimal combination of process parameters, with the aim of minimizing surface roughness and flank wear. Pawar and Rao (2013) applied the teaching–learning-based optimization (TLBO) algorithm to optimize the process parameters of abrasive water jet machining, grinding, and milling. They created physics-based models between the input and output parameters of each process and then utilized TLBO to minimize the material removal rate in abrasive water jets, minimize production cost and maximize production rate with respect to grinding, and minimize the production time in milling. Fard et al. (2013) employed adaptive network-based fuzzy inference systems (ANFIS) to model the process of dry wire electrical discharge machining (WEDM). This model was then used to optimize, using artificial bee colony (ABC), the process inputs that would minimize surface roughness and maximize material removal rate. Teixidor et al. (2013) used particle swarm optimization (PSO) to obtain optimal process parameters that would minimize the depth error, width error, and surface roughness in the pulsed laser milling of micro-channels on AISI H13 tool steel. Katherasan et al. (2014) used ANN to model the process of flux cored arc welding (FCAW) and then utilized PSO to minimize bead width and reinforcement and maximize depth of penetration. Yusup et al. (2014) created a regression model for the process parameters and process indicators of an abrasive waterjet (AWJ) and then used ABC to minimize the surface roughness. Panda and Yadava (2012) used ANN to model the process of die sinking electrochemical spark machining (DS-ESM) and then used GA for multi-objective optimization of the material removal rate and average surface roughness. Maji and Pratihar (2010) combined ANFIS with GA to create forward and backward input–output relationships for the electrical discharge machining process (EDM). In their proposed methodology, GA was used to optimize the membership functions of the ANFIS, with the aim of minimizing the error between the predicted and actual outputs. Cus et al. (2006) developed an intelligent system for online monitoring and optimization of process parameters in the ball-end milling process. Their objective was to find the optimal set of process parameters, using GA to achieve the forces selected by the user. Raja et al. (2015) optimized the process parameters of electric discharge machining (EDM) using the firefly algorithm to obtain the desired surface roughness in the minimum possible machining time. Raja and Baskar (2012) used PSO to optimize the process parameters to achieve the desired surface roughness while minimizing machining time for face milling. Rao and Pawar (2009) developed mathematical models using response surface modeling (RSM) to correlate the process inputs and performance indicators of WEDM. They then used ABC to achieve the maximum machining speed that would give the desired value of the surface finish. Lee et al. (2007) modeled the process of high-speed finish milling using a 2 stage ANN and then used GA to maximize the surface finish while achieving the desired material removal rate. Teimouri and Baseri (2015) used a combination of fuzzy logic and the artificial bee colony algorithm to create a forward prediction model between input and output parameters for friction stir welding (FSW). This trained model was then utilized to find the optimal input parameters that would give the desired output value by minimizing the absolute error between the predicted and specified output using the imperialist competitive algorithm (ICA).

An ample amount of work has also been done to create a reverse mapping model between the process parameters and the performance indicators. Parappagoudar et al. (2008) utilized the back-propagation neural network (BPNN) and a genetic-neural network (GA-NN) for forward and reverse mapping of the process parameters and performance indicators in a green sand mold system. Parappagoudar et al. (2008) also extended their application of BPNN and GA-NN to create forward and backward mappings for the process of the Sodium Silicate-Bonded, Carbon Dioxide Gas Hardened Molding Sand System. Amarnath and Pratihar (2009) used radial basis function neural networks (RBFNNs) for forward and reverse input–output mapping of the tungsten inert gas (TIG) welding process. In their proposed methodology, the structure and the parameters of the RBFNN were modified using a combination of GA and the fuzzy C-means (FCM) algorithm for both the forward and reverse mapping. Chandrashekarappa et al. (2014) used BPNN and GA-NN for forward and reverse mappings of the squeeze casting process. Kittur and Parappagoudar (2012) utilized BPNN and GA-NN for forward and reverse mapping in the die casting process. Because batch training requires a tremendous amount of data, they used previously generated equations to supplement the experimental data. Malakooti and Raman (2000) used ANN to create forward- and backward-direction mappings between the process outputs and inputs for the cutting operation on a lathe.

Even though extensive research has been done regarding optimization of the process parameter for different processes, the current algorithms used for the optimization procedure are limited to finding only one set of optimal process parameter combinations for a single-objective optimization problem each time the algorithms are executed. Though this process parameter combination may achieve the desired output, it may not always be suitable for actual production or may lead to undesirable experimental conditions. It can also be observed from the experimental data of different processes that different process parameter combinations may lead to the same or similar performance indicators. For example, in turning, multiple combinations of process parameters may lead to the same or similar value of surface roughness. In EMM, multiple combinations of process parameters may lead to the same or similar value of taper and overcut. Therefore, there is a possibility to develop a method that can provide multiple optimal process parameter combinations for a single-objective optimization problem.

In this paper, the presented method is to obtain multiple optimal process parameter combinations for a single-objective optimization problem by splitting the original search space into smaller sub-search spaces and finding the optimal process parameter combinations in each sub-search space. Two different methods are used to split the original search space, and GA is utilized to optimize the process parameters in each sub-search space. The optimization results obtained after using the two search space splitting methods are compared to the optimization results obtained when the original search space was divided equally into smaller sub-search spaces; GA was used to optimize the process parameters in each sub-search space. EMM of SUS 304 is used as a case study because its experimental data shows that multiple process parameter combinations can lead to the same performance indicators. Due to the lack of physics-based models, a general regression neural network (GRNN) is used to create a forward prediction model between the input process parameters and the performance indicators for the process of EMM. The rest of the paper is organized as follows: section “Modeling” describes the modeling stage of the method. Section “Case study” presents and discusses the results obtained. Section “Conclusion” presents conclusions from the presented work and mentions future directions for the proposed approach.

## Modeling

### Split-optimization approach

**Fig. 1:**
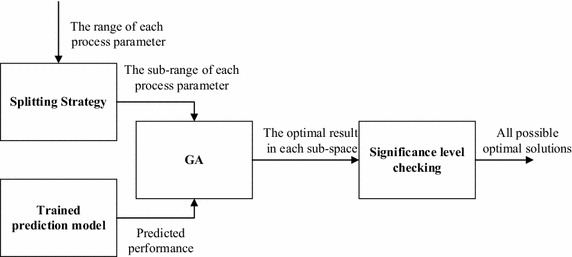
The basic structure of the split-optimization approach

Traditional GA, when used in a single-objective optimization, only converges to a single local optima or near-optimum solution, while the search space might consist of multiple local optima that can satisfy the given criteria. Multi-objective GA, on the other hand, does provide multiple solutions, but each solution satisfies each objective to a different degree. A possible method to obtain multiple solutions for a single-objective optimization problem is to split the original search space into several smaller sub-search spaces, with each sub-search space containing a possible solution to the given objective. Once these sub-search spaces have been identified, GA can then be used in each sub-search space to find the possible solution. The procedure of the proposed split-optimization approach consists of two parts: splitting of the original search spaces into sub-search spaces and the application of GA to find the solution in each sub-search space. Because any optimization function needs a fitness function as an input, in this paper, a generalized neural network (GRNN) was used as the fitness function due to a lack of physics-based models for the given process. The flow chart of this proposed split-optimization approach is shown in Fig. 1.

Because the results obtained after using GA depend on the training accuracy of the GRNN, it is important to train the GRNN sufficiently so that it can predict the performance indicators with a high degree of accuracy. As there will always be some degree of error associated with the outputs of the GRNN, a possible method to cope with these errors is to take into consideration the significance level of the optimization problem. The significance level here is defined as a customized parameter that allows solutions with a fitness value better than or equal to it to be counted as final optimal solutions. The significance level by default is regarded as zero, which indicates that only solutions with the same minimum fitness value can be regarded as the final optimum solutions.

### Splitting strategies

As mentioned earlier, two strategies are used to split the original search space into sub-search spaces. The details of the two strategies are highlighted below.

1. *Hill and valley splitting strategy*

The steps of the splitting strategy are as follows:

- Identify two data points, ***A*** and ***B***, from the experimental data set whose input values are furthest away from each other. Here, $A = \left( {a_{1} ,a_{2} , \cdots ,a_{n} ,y_{a} } \right)$ and $B = \left( {b_{1} ,b_{2} , \cdots ,b_{n} ,y_{b} } \right)$, indicating that all the data points have *n* inputs and 1 output.
- Select a random data point ***C***_***1***_ from the remaining data points and determine whether it is a hill, valley, or neither compared to the initial points, i.e., ***A*** and ***B***, based on the value of its output. For example, if $y_{a}$ < $y_{b}$ < $y_{{c_{1} }}$, then ***C***_***1***_ is a hill; if $y_{{c_{1} }}$ < $y_{a}$ < $y_{b}$, then ***C***_***1***_ is a valley; if $y_{a}$ < $y_{{c_{1} }}$ < $y_{b}$, then ***C***_***1***_ is neither.
- Select a random data point ***C***_***2***_ from the remaining data points; find a pair of previously selected data points whose input values encompass the input values of ***C***_***2***_.
- Compare the output value of ***C***_***2***_ with the data points selected in step c and determine whether it is a hill, valley, or neither.
- Repeat step c and d until all the data points have been identified as a hill, valley, or neither.

**Fig. 2:**
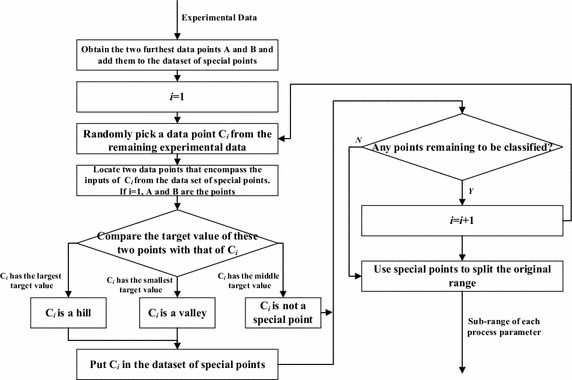
The flow chart of the *hill and valley splitting strategy*

After the classification of all the experimental data points is completed, the input values of the original points (***A*** and ***B)*** and all the points classified as either a hill or valley are used to split the original search space into smaller sub-search spaces. This is done by dividing the original range of the input parameters of the experimental data into sub-ranges by using the input values of the points classified as hill or valley and then finding all the combinations of the sub-ranges for all the inputs. Once the search space has been split into sub-search spaces, GA is used to optimize each search space individually. Figure 2 shows the flow chart of the *hill and valley splitting strategy*.

(2) *Cluster centers splitting strategy*

**Fig. 3:**
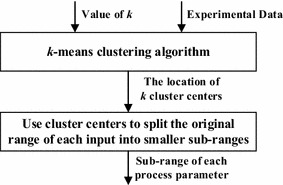
The flow chart of the *cluster centers splitting strategy*

In this strategy, the *k*-*means clustering algorithm* is used to divide the experimental data set into *k* clusters. Once the *k* cluster centers are identified, they are used to split the original search space into smaller sub-search spaces. This is accomplished by dividing the original range of input process parameters of the experimental data into smaller sub-ranges using the values of the *k* cluster centers. Next, the original search space is divided by using all the combinations of the sub-ranges for all the inputs. Figure 3 shows the flow chart of the *cluster centers splitting strategy*.

### GRNN-GA optimization

As mentioned earlier, a forward prediction model was created using GRNN (Specht 1991). The inputs of the GRNN were voltage, pulse on time, and feed rate; the outputs were *D*_*in*_ and *D*_*out*_. During the training of the GRNN, the original data was split into training, validation, and testing data sets, and tenfold cross validation was used during the training phase of the GRNN to avoid overfitting and to find the optimal value of the spread parameter that would minimize the mean squared error (MSE). Once the GRNN was trained sufficiently, it was then utilized as the fitness function for GA during the optimization procedure.

## Case study

An input parameter optimization problem in EMM was utilized as a case study because it can be seen from Table 1 that multiple combinations of input process parameters lead to the same or similar values of taper and overcut.

**Table 1:** Original range of the controllable process parameters

Process parameter	Voltage (V)	Pulse on time (µs)	Feed rate (µm/s)
Lower bound	8	25	4
Upper bound	20	70	12

### Description of the case

**Fig. 4:**
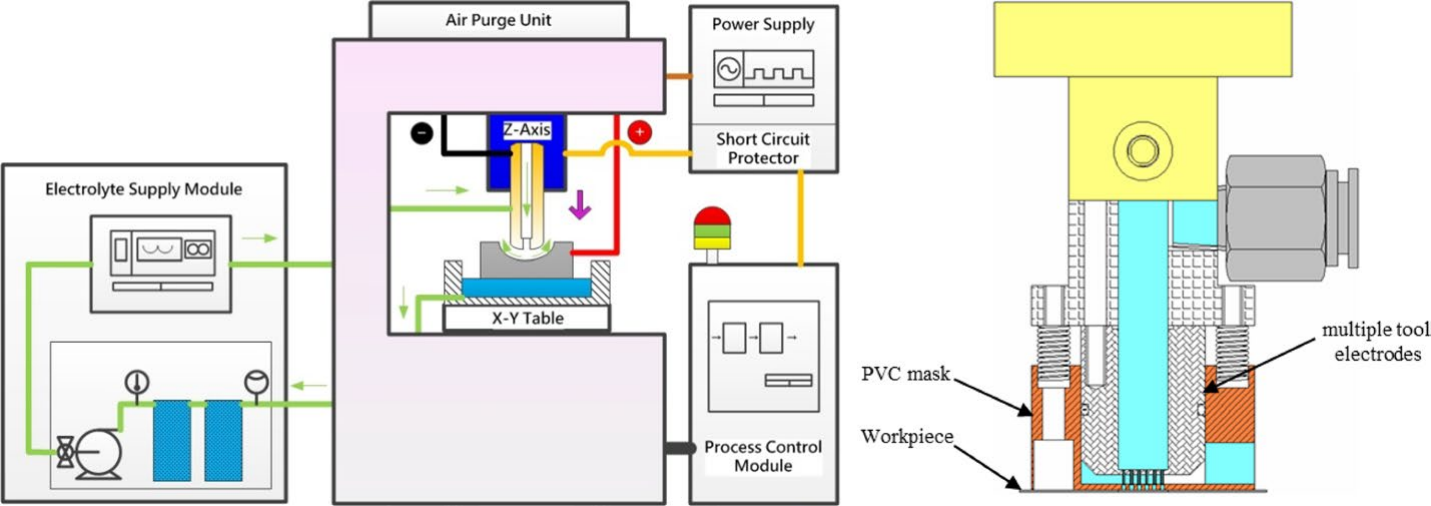
Schematic diagram of electrochemical micromachining system (*left*) and micro array hole electrode module (*right*)

Figure 4 schematically depicts the EMM experimental setup. The system consists of a three-dimensional movement device, a small-scale power supply of 100 A, and an electrolyte pump and filter. The feeding system is controlled by a PC-Based CNC Controller, RTX real-time windows kernel program, and a motion card that drives the linear motor precisely. A pulse generator supplies a periodic current to the experimental model. A digital oscilloscope ensures that the pulse generator produces a rectangular waveform with accurate amplitude. If the tool feed rate is excessive, the tool will contact the workpiece and cause a short circuit; thus, an oscilloscope is employed to detect any short circuits. Whenever the oscilloscope detects a short circuit, a signal is sent rapidly to the PC and the tool is extracted automatically until the measured voltage returns to the applied voltage. The micro array holes electrode module includes the multiple nozzle tool electrodes, PVC mask and tool fixture. The electrolyte is pumped to a multiple electrode cell and exits through the small nozzle in the form of a free standing jet directed towards the anode workpiece.

Other basic information and settings are as follows: the electrolyte velocity was 10 m/s, electrolyte temperature was 27 °C, the initial gap between the tool and the workpiece was 100 µm, tool moving distance was 800 µm, the workpiece material was SUS 304, the electrolyte used was 10 %wt. NaNO_3_, the nominal diameter of the hole was 900 µm, and the depth of the hole was 500 µm.

Voltage, pulse on time, and feed rate were used as the controllable process parameters, while the inner diameter of the micro-hole *D*_*in*_ and the outer diameter *D*_*out*_ were the measurable performances. The range of each process parameter is shown in Table 1. The range of the variables was fixed by taking into consideration two factors: 1. limitation of the devices used for EMM and 2. making sure that the experimental conditions would be stable within the chosen range. The resolution of the process parameters were was 0.1 V for the voltage, 0.1 µs for pulse on time, and 0.1 µm/s for the feed rate. This indicates that there are close to 3 million possible combinations of all the process parameters. Therefore, the proposed method was applied for this particular case study.

The process of EMM has two responses, i.e., taper and overcut. When drilling micro-size holes in thin metallic foils, a major requirement is for the holes to have straight walls. The straightness of a wall can be represented by the taper and is given by:

1 $$Taper = \left| {(D_{in} - D_{out} )/depth_{{}} } \right|$$

In critical applications, particularly in micro instruments, the straightness of a drilled hole is also very important. Overcut, as given by Eq. (2), is the difference between the aim holes’ diameters and actual hole diameter and is a good representation of the straightness of a drilled hole. A small overcut value represents a more precise EMM process.

2 $$Overcut = \left| {(D_{in} - D)/2_{{}} } \right|$$

In the process of EMM, the aim is to find the set of process parameter combinations that will minimize both taper and overcut. Though EMM has two responses, for the purpose of this case study, the two responses were combined into a single-objective by the use of weight values. Before combining them into a single objective, the values of taper and overcut were normalized between 0 and 1. Equation (3) shows how the taper and overcut were normalized, while Eq. (4) shows the objective function.

3 $$T_{{normalized}} = \frac{{T_{{predicted}} - T_{{\min ,experimental}} }}{{T_{{\max ,experimental}} - T_{{\min ,experimental}} }},O_{{normalized}} = \frac{{O_{{predicted}} - O_{{\min ,experimental}} }}{{O_{{\max ,experimental}} - O_{{\min ,experimental}} }}$$

where $T_{predicted}$ is the taper value predicted by the GRNN, $T_{{\min} ,experimental}$ is the minimum taper value in the experimental data, $T_{{\max} ,experimental}$ is the maximum taper value in the experimental data, and $T_{normalized}$ is the normalized predicted taper value. Similarly, $O_{predicted}$ is the overcut value predicted by the GRNN, $O_{{\rm min} ,exp erimental}$ is the minimum overcut value in the experimental data, $O_{{\rm max} ,exp erimental}$ is the maximum overcut value in the experimental data, and $O_{normalized}$ is the normalized predicted overcut value.

4 $$Objective = 0.5 \times T_{normalized} + 0.5 \times O_{normalized}$$

To create a forward prediction model for the process of EMM, three different sets of experiments were created. In the first experimental set, voltage and feed rate had 3 levels each, while pulse on time was constant, which resulted in a total of 9 combinations of input parameters. These combinations of input experiments were used to perform the process of EMM, and for each combination, *D*_*in*_ and *D*_*out*_ were recorded. In the second and third experimental sets, voltage, pulse on time, and feed rate had 3 levels each, which resulted in 27 combinations of input process parameters for both experimental sets 2 and 3. The process of EMM was performed using the combination of inputs; *D*_*in*_ and *D*_*out*_ were again recorded. The levels of voltage, pulse on time, and feed rate are given in Table 2.

**Table 2:** Levels of voltage, pulse on time, and feed rate values used for the three experimental sets

Experimental set #	Levels of voltage (V)	Levels pulse on time (µs)	Levels feed rate (µm/s)
1	[16, 18, 20]	25	[4, 6, 8]
2	[4, 6, 8]	[50, 60, 70]	[8, 10, 12]
3	[4, 6, 8]	[50, 60, 70]	[8–10]

**Fig. 5:**
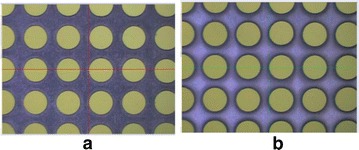
Pictures taken using the CCD camera. **a** The front of the workpiece, while **b**. The back of the workpiece

In the experiments, the Charge Coupled Device (CCD) camera was utilized to measure all the workpieces after the process of EMM. Figure 5 shows the pictures taken using the CCD camera. The CCD images were then processed through a software, which provided the average value of the diameters of the holes on the front and back of the workpiece. The experimental data obtained is shown in Table 3.

**Table 3:** The 63 groups of experimental data

No.	Voltage (V)	Pulse on time (µs)	Feed rate (µm/s)	*D*_*in*_ (µm)	*D*_*out*_ (µm)	Taper	Overcut (µm)
1	16	25	8	893	860	0.066	3.5
2	18	25	8	929	913	0.032	14.5
3	20	25	8	923	910	0.026	11.5
4	16	25	6	904	892	0.024	2
5	18	25	6	934	931	0.006	17
6	20	25	6	999	977	0.044	49.5
7	16	25	4	983	979	0.008	41.5
8	18	25	4	1050	1045	0.01	75
9	20	25	4	1125	1123	0.004	112.5
10	8	50	8	657.5	627.5	0.06	121.25
11	10	50	8	809.5	807.25	0.0045	45.25
12	12	50	8	866.25	858	0.0165	16.875
13	8	50	6	760	741	0.038	70
14	10	50	6	828.5	829.5	0.002	35.75
15	12	50	6	908.75	905.5	0.0065	4.375
16	8	50	4	781.75	780.25	0.003	59.125
17	10	50	4	887.25	881.75	0.011	6.375
18	12	50	4	957.75	970	0.0245	28.875
19	8	60	8	771.33	759.33	0.024	64.335
20	10	60	8	806.75	799.5	0.0145	46.625
21	12	60	8	862.75	847	0.0315	18.625
22	8	60	6	756.5	739.75	0.0335	71.75
23	10	60	6	776.75	777.5	0.0015	61.625
24	12	60	6	840.25	841.25	0.002	29.875
25	8	60	4	769	771.5	0.005	65.5
26	10	60	4	854.75	865.25	0.021	22.625
27	12	60	4	928.25	945.5	0.0345	14.125
28	8	70	8	718	721.5	0.007	91
29	10	70	8	779	796.75	0.0355	60.5
30	12	70	8	841.5	849.75	0.0165	29.25
31	8	70	6	736.5	744.5	0.016	81.75
32	10	70	6	802	829.75	0.0555	49
33	12	70	6	858.75	865	0.0125	20.625
34	8	70	4	783.25	783.25	0	58.375
35	10	70	4	878.75	872	0.0135	10.625
36	12	70	4	946.25	955.25	0.018	23.125
37	8	50	8	874	704	0.34	13
38	9	50	8	914	789	0.25	7
39	10	50	8	999	827	0.344	49.5
40	8	50	6	922	765	0.314	11
41	9	50	6	955	807	0.296	27.5
42	10	50	6	1039	837	0.404	69.5
43	8	50	4	932	797	0.27	16
44	9	50	4	1044	790	0.508	72
45	10	50	4	1130	858	0.544	115
46	8	60	8	903	708	0.39	1.5
47	9	60	8	967	766	0.402	33.5
48	10	60	8	1084	817	0.534	92
49	8	60	6	917	760	0.314	8.5
50	9	60	6	1043	856	0.374	71.5
51	10	60	6	1115	871	0.488	107.5
52	8	60	4	1071	754	0.634	85.5
53	9	60	4	1087	972	0.23	93.5
54	10	60	4	1263	1044	0.438	181.5
55	8	70	8	875	789	0.172	12.5
56	9	70	8	1071	842	0.458	85.5
57	10	70	8	1158	862	0.592	129
58	8	70	6	987	846	0.282	43.5
59	9	70	6	1212	886	0.652	156
60	10	70	6	1243	1056	0.374	171.5
61	8	70	4	1134	877	0.514	117
62	9	70	4	1260	935	0.65	180
63	10	70	4	1348	1016	0.664	224

## Results

As stated earlier, to compensate for the errors associated with the trained GRNN, a significance level needs to be specified. In this case study, if the value of the objective function, given by Eq. (4), after optimization was less than 0.5, then the solution of that particular sub-search space was said to be a final optimal solution. The only changeable parameter for the GRNN was the spread value, which was obtained after the training process. The changeable parameters for GA are listed in Table 4.

**Table 4:** Parameter values used for GA

Number of generations	Population size	Crossover fraction	Mutation fraction	Elite count
100	50	0.85	0.15	3

The methods mentioned above were used to split and optimize the search space 10 times independently, and the average value of the objective function for the best solutions of each run was calculated. The run that had the lowest average value of the objective function was used as the best run; its results are presented here.

1. *Hill and valley spitting strategy*

As mentioned previously, the first step in this method is to find two data points that are furthest away from each other. To accomplish this task, the distance from the origin to every data point was obtained after each input was normalized using Eq. (6). The equations used to normalize the inputs are given in Eq. (5). The two data points with distances *d*_*min*_ and *d*_*max*_ were the inputs furthest away from each other. Then, the steps outlined in the previous section were followed to split the original search space into several sub-search spaces.

5 $$V_{i,normalized} = \frac{{V_{i} - V_{{\rm min} ,exp erimental} }}{{V_{{\rm max} ,\exp erimental} - V_{{\rm min} ,exp erimental} }},\;P_{i,normalized} = \frac{{P_{i} - P_{{\rm min} ,exp erimental} }}{{P_{{\rm max} ,exp erimental} - P_{{\rm min} ,exp erimental} }},\;F_{i,normalized} = \frac{{F_{i} - F_{{\rm min} ,exp erimental} }}{{F_{{\rm max} ,exp erimental} - F_{{\rm min} ,exp erimental} }}$$

where $V_{i,normalized}$ is the normalized values of voltage in the *i*th experimental data, $V_{{\rm min} ,exp erimental}$ is the minimum voltage value in the experimental data, $V_{{\rm max} ,exp erimental}$ is the maximum voltage value in the experimental data, and $V_{i}$ is the voltage in the *i*th experimental data. Similarly, $P_{i,normalized}$ is the normalized values of pulse on time in the *i*th experimental data, $P_{{\rm min} ,exp erimental}$ is the minimum pulse on time value in the experimental data, $P_{{\rm max} ,exp erimental}$ is the maximum pulse on time value in the experimental data, and $P_{i}$ is the pulse on time in the *i*th experimental data. $F_{i,normalized}$ is the normalized values of feed rate time in the *i*th experimental data, $F_{{\rm min} ,exp erimental}$ is the minimum feed rate value in the experimental data, $F_{{\rm max} ,\exp erimental}$ is the maximum feed rate value in the experimental data, and $F_{i}$ is the feed rate in the *i*th experimental data.

6 $$D_{i} = \sqrt {\left( {V_{i,normalized} } \right)^{2} + (P_{i,normalized} )^{2} + (F_{i,normalized} )^{2} }$$

Table 5 provides the ranges for each of the input values. For each of the sub-search spaces, GA was utilized to find the optimal process parameter combination. The optimization results are shown in Table 6.

**Table 5:** Splitting result of the *hill and valley splitting strategy*

Number of hills	Number of valleys	Sub-range of voltage (V)	Sub-range of pulse on time (µs)	Sub-range of feed rate (µm/s)	Number of sub-search spaces
29	14	[8, 9]; [9, 10]; [10, 18]; [18, 20]	[25,50]; [50,60]; [60,70]	[4, 6]; [6, 8]	24

**Table 6:** Optimization results obtained after using the *hill and valley splitting strategy*

No.	Voltage (V)	Pulse on Time (µs)	Feed rate (µm/s)	*D*_*in*_ (µm)	*D*_*out*_ (µm)	Taper	Overcut (µm)
1	18.0	60.0	6.0	901.05	892.68	0.02	0.52
2	19.4	60.0	5.9	900.00	895.82	0.01	0.00
3	18	61.6	6.0	901.59	894.02	0.02	0.80
4	19.9	67.1	5.9	900.00	898.96	0.00	0.00
5	18.0	60.0	6.2	900.43	891.84	0.02	0.21
6	19.6	59.6	6.1	900.00	895.94	0.01	0.00
7	18.0	62.6	6.3	901.38	893.94	0.01	0.69
8	20.0	68.6	6.1	900.00	899.24	0.00	0.00

(2) *Cluster centers splitting strategy*

The *k* value in the *k*-*means clustering algorithm* is a user dependent parameter; an inappropriate choice of *k* may yield poor results. However, so far there is no clear guideline for choosing the value of *k*. In this case study, the value of k was varied from 2 to 6; the corresponding splitting and optimization results are shown in Table 7. The maximum number of optimal solutions was obtained when the value of *k* was 6. These results are shown in Table 8.

**Table 7:** Splitting result obtained using *cluster centers strategy*

Value of *k*	Cluster centers	Sub-range of voltage (V)	Sub-range of pulse on time (µs)	Sub-range of feed rate (µm/s)	Number of sub-spaces
2	[10 *V*,60 µs,6 µm/s]; [18 *V*,25 µs,6 µm/s]	[8,10]; [10,18]; [18, 20]	[25, 60]; [60,70]	[4, 6]; [6, 8]	12
3	[10 *V*,55 µs,6 µm/s]; [10 *V*,70 µs,6 µm/s]; [18 *V*,25 µs,6 µm/s]	[8,10]; [10,18]; [18,20]	[25,55]; [55,70]	[4, 6]; [6, 8]	12
4	[10 *V*,60 µs,6 µm/s]; [10 *V*,70 µs,6 µm/s]; [18 *V*,25 µs,6 µm/s]; [10 *V*,50 µs,6 µm/s]	[8,10]; [10, 18]; [18,20]	[25,50]; [50,60]; [60,70]	[4,6]; [6,8]	18
5	[9 *V*,50 µs,5 µm/s]; [10 *V*,70 µs,6 µm/s]; [18 *V*,25 µs,6 µm/s]; [10 *V*,60 µs,6 µm/s]; [10 *V*,50 µs,8 µm/s]	[8, 9]; [9, 10]; [10, 18]; [18, 20]	[25,50]; [50,60]; [60,70]	[4,5]; [5,6]; [6, 8]	36
6	[9 *V*,50 µs,5 µm/s]; [10 *V*,50 µs,8 µm/s]; [10 *V*,70 µs,7 µm/s]; [9 *V*,70 µs,5 µm/s]; [10 *V*,60 µs,6 µm/s]; [18 *V*,25 µs,6 µm/s]	[8, 9]; [9, 10]; [10, 18]; [18, 20]	[25,50]; [50,60]; [60,70]	[4, 5]; [5, 6]; [6, 7]; [7, 8]	48

**Table 8:** The optimization results obtained using the *cluster centers splitting strategy* with *k* = 6

No.	Voltage (*V*)	Pulse on time (µs)	Feed rate (µm/s)	*D*_*in*_ (µm)	*D*_*out*_ (µm)	Taper	Overcut (µm)
1	18.0	59.7	6.0	901.01	892.49	0.02	0.50
2	19.2	59.1	5.9	900.00	894.85	0.01	0.00
3	18.0	61.6	6.0	901.58	893.98	0.02	0.79
4	19.9	67.6	6.0	900.00	899.03	0.00	0.00
5	18.0	60.0	6.2	900.44	891.85	0.02	0.22
6	19.8	60.0	6.2	900.00	895.87	0.01	0.00
7	18.0	61.7	6.2	901.00	893.27	0.02	0.50
8	20.0	68.1	6.0	900.00	899.19	0.00	0.00
9	18.0	70.0	7.5	900.00	890.99	0.02	0.00
10	18.9	70.0	7.0	899.97	895.46	0.01	−0.01

(3) Equally splitting strategy

The results obtained using the two previous splitting strategies were compared to the results obtained when the original search space was split equally into smaller sub-search spaces. In the equally splitting strategy, each process parameter was equally split into 4 sub-ranges, as shown in Table 9. The optimization results obtained using the equally splitting strategy are shown in Table 10.

**Table 9:** Splitting result of equally splitting strategy

Sub-range of voltage (V)	Sub-range of pulse on time (µs)	Sub-range of feed rate (µm/s)	Number of sub-spaces
[8, 11]; [11, 14]; [14, 17]; [17, 20]	[25,36]; [36,47]; [47,58]; [58,70]	[4, 5]; [5, 6]; [6, 7]; [7, 8]	64

**Table 10:** The optimization result obtained using the equally splitting strategy

No.	Voltage (V)	Pulse on time (µs)	Feed rate (µm/s)	*D*_*in*_ (µm)	*D*_*out*_ (µm)	Taper	Overcut (µm)
1	19.1	58.8	6.0	900.01	894.33	0.01	0.01
2	20.0	65.8	5.8	900.00	898.78	0.00	0.00
3	19.2	58.7	6.1	900.00	894.42	0.01	0.00
4	20.0	69.4	6.3	900.00	899.29	0.00	0.00
5	18.8	70.0	7.0	899.98	895.45	0.01	−0.01

### Comparison and analysis

These three splitting strategies provide different ways to split the search space into smaller sub-search spaces. To evaluate the efficiency of a strategy, the percentage of useful sub-search spaces was calculated using Eq. (7).

7 $$percentage\,of\, useful\, sub\,- search\, spaces = \frac{{No.\,of\, optimal\, solutions}}{{No.\, of\, sub - search\, spaces}} \times 100\,\%$$

Table 11 shows the comparison between the 3 strategies based on Eq. (7).

**Table 11:** Comparison of three splitting strategies

Name of splitting strategy	*Hill and valley splitting*	*Cluster centers splitting*					Equally splitting
Value of parameter	–	*k* = 2	*k* = 3	*k* = 4	*k* = 5	*k* = 6	4
No. of sub-spaces	24	12	12	18	36	48	64
No. of solutions	8	8	4	8	8	10	5
Percentage of useful sub-spaces	33.3 %	66.7 %	33.3 %	44.4 %	22.2 %	20.8 %	7.8 %

It can be observed that the equally splitting strategy is the least efficient way because its percentage of useful sub-search spaces is the lowest (7.8 %). The efficiency of *hill and valley splitting* is fixed because it lacks any controllable parameters and because the sequence in which points are selected can affect their classification. It can be seen from Table 10 that there is a correlation between the efficiency of *cluster centers splitting* and the value of *k*. However, there is no clear understanding between the value of k and the efficiency of the method and there is also no guideline for selecting the optimal value of *k.*

This case study utilized a trained NN prediction model in the evaluation of input parameter combinations. Therefore, to validate the optimization result, one additional experiment with the randomly chosen optimized input parameter combination was done. The data of the validation experiment is shown in Table 12.

**Table 12:** Result of an additional validation experiment

	Voltage (V)	Pulse on time (µs)	Feed rate (µm/s)	*D*_*in*_ (µm)	*D*_*out*_ (µm)	Taper	Overcut (µm)
Predicted	19.2	59.1	6	900.00	894.85	0.01	0.00
Experimental				899	894	0.01	0.00

Based on the validation experimental result, it can be seen that the prediction error of the NN prediction model used in this case study is quite low and the results obtained using the proposed approach are better than the results shown in the initial experimental data. Noteworthy, the optimized input process parameter combination was not in the initial training dataset and the optimization algorithm was able to find a better-than-ever objective value. Therefore, the optimization result is verified.

## Conclusion

In this paper, a split-optimization approach was proposed for obtaining multiple solutions for a single-objective process parameter optimization problem. The proposed approach consisted of two stages: splitting of the original search space into smaller sub-search spaces and optimization of process parameters in each of the smaller sub-search spaces. Two splitting strategies, i.e., *hill and valley splitting strategy* and *cluster centers splitting strategy*, were used to split the original search space into smaller sub-search spaces efficiently. Next, GA was used in each sub-search space to find multiple combinations of process parameters that minimized the single-objective value, one from each sub-search space. The efficiency of these two strategies was verified by comparing them with a method in which the original search space is divided into smaller and equal sub-search spaces. The comparison of the results from the different splitting methods showed that the *hill and valley splitting strategy* and *cluster centers splitting strategy* were more efficient than the equal splitting strategy. Among all the methods, the *cluster centers splitting strategy*, for a *k* value of 6, was able to achieve the most optimal solutions. The results obtained from the *hill and valley splitting strategy* showed that though it is an efficient method, its efficiency depends on the order in which the points are classified as a hill or valley.

Possible future work includes a study of the relationship between the efficiency of the *cluster centers splitting strategy* and the *k* value; a guideline should be to choose an optimal value of *k*. Future works also include experimentally validating the multiple solutions obtained using the proposed approach, applying the proposed approach to more case studies, and refining the proposed approach based on the results of the experimental validation and other case studies.
